# Newly discovered mutations in the GALNT3 gene causing autosomal recessive hyperostosis-hyperphosphatemia syndrome

**DOI:** 10.1080/17453670902807482

**Published:** 2009-02-01

**Authors:** Faysal Gok, Ilana Chefetz, Margarita Indelman, Murat Kocaoglu, Eli Sprecher

**Affiliations:** ^1^Department of Pediatric Nephrology and Radiology, Gulhane Military Medical SchoolAnkaraTurkey; ^2^Radiology, Gulhane Military Medical SchoolAnkaraTurkey; ^3^Center for Translational Genetics, Rappaport Institute for Research in the Medical Sciences, Israel Institute of TechnologyHaifaIsrael; ^4^Laboratory of Molecular Dermatology, Department of Dermatology, Rambam Health Care CampusHaifaIsrael; ^5^Bruce Rappaport Faculty of MedicineHaifaIsrael

## Abstract

**Background and purpose** Periosteal new bone formation and cortical hyperostosis often suggest an initial diagnosis of bone malignancy or osteomyelitis. In the present study, we investigated the cause of persistent bone hyperostosis in the offspring of two consanguineous parents.

**Methods** Clinical assessment, imaging, and direct sequencing were used to elucidate the etiology of the condition seen in the patient.

**Results** Radiological examination revealed periosteal reaction, diaphysitis, and cortical hyperostosis, suggesting osteomyelitis or a bone neoplasm. The clinical and radiological features were also reminiscent of hyperostosis with hyperphosphatemia (HHS), a rare autosomal recessive disease manifesting with recurrent, transient, and painful swelling of the long bones. The identification of two novel heterozygous pathogenic mutations in the GALNT3 gene confirmed a diagnosis of HHS.

**Interpretation** Molecular analysis represents an invaluable tool in the differential diagnosis of persistent cortical hyperostosis.

## Introduction

Hyperostosis with hyperphosphatemia (HHS; Mendelian Inheritance in Man (MIM) 610233) is an extremely rare metabolic disorder, characterized by repeated painful swelling of long bones accompanied by radiological findings of diaphysitis such as cortical hyperostosis and periosteal new bone formation. These unspecific clinical and radiological findings may lead to delay in diagnosis and unnecessary and invasive diagnostic procedures (Mikati et al. 1981, Clarke et al. 1984, Narchi 1997).

Hyperphosphatemic familial tumoral calcinosis (HFTC; MIM211900) is another rare recessive disorder characterized by the development of large periarticular calcified masses, often associated with painful and mutilating skin ulcerations (Metzker et al. 1988). Both HFTC and HSS are associated with marked and persistent hyperphosphatemia resulting from increased renal tubular reabsorption of phosphate (Liu and Quarles 2007); the two diseases have rarely been reported in the same family (Narchi 1997). Accordingly, both conditions have been found to result from mutations in the same gene, GALNT3, which encodes the enzyme UDP-N-acetyl-alpha-D-galactosamine:polypeptide N-acetylgalactosaminyltransferase 3 (ppGalNacT3) (Topaz et al. 2004, Frishberg et al. 2005). ppGalNacT3 has been found to mediate O-glycosylation of FGF23 (Frishberg et al. 2007). FGF23 activity is regulated through the activity of a number of proteases that convert active FGF23 into two inactive proteolytic fragments (Liu and Quarles 2007). ppGalNacT3-mediated O-glycosylation is thought to protect FGF23 from proteolysis (Frishberg et al. 2007) and to be necessary for proper secretion of FGF23 (Kato et al. 2006).

We describe a patient with HSS, initially diagnosed as chronic osteomyelitis. This study underscores the complexity of the differential diagnosis of cortical hyperostosis and the usefulness of non-invasive molecular diagnostics in such cases.

## Patients and methods

We studied a family of Turkish origin. All participants or their legal guardian provided written and informed consent according to a protocol previously approved by the local institutional review board. 15 mL of blood was drawn from each subject, and DNA was extracted using a salt/chloroform extraction method.

### Mutational analysis

All exons and exon-intron boundaries of the GALNT3 and FGF23 genes were amplified by PCR as previously described (Benet-Pages et al. 2005, Frishberg et al. 2005). PCR amplification was performed using Taq polymerase and Q solution according to the manufacturer’s instructions (Qiagen, Valencia, CA). Gel-purified amplicons were subjected to bidirectional sequencing using Big Dye Terminator (PE Applied Biosystems, Foster City, CA).

### PCR-RFLP

To verify mutation c.T2A (see Mutation Analysis below), a 187-bp PCR fragment encompassing part of exon 1 was amplified using primers 5’- GTAGGACTGAATAGCTACTAATAC-3’ and 5’- GTGTAATTTTACTAGTCGCTTTAGGTGAGGC -3’, and digested in the presence of BsmI. To verify mutation c.G839A, a 470-bp PCR fragment encompassing exon 4 was amplified using primers 5’- CAATAAATCTGAGGAAGAAAGAAATC-3’ and 5’- GTACACACTGTTTGCTTTATAGC-3’, and digested in the presence of BstOI.

## Results

### Clinical findings

**Figure 1.**
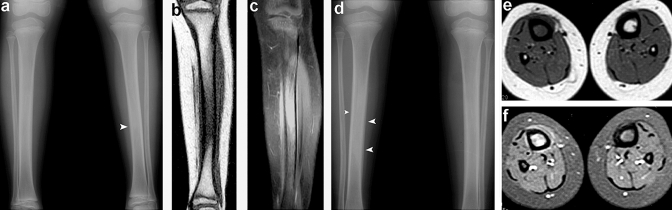
(a) Radiograph demonstrating a subtle periosteal reaction in the midshaft of the left tibia (arrowhead). (b) A coronal T1-weighted sequence showing loss of normal fatty marrow in the tibia. (c) A post-contrast fat-suppressed coronal T1-weighted image revealing contrast enhancement in the marrow and adjacent soft tissues. (d) Radiograph showing a periosteal reaction (arrowheads) and medullary sclerosis of the right tibia. (e) An axial T1-weighted image demonstrating reduced bone marrow signal of the right tibia with adjacent soft tissue intensity. (f) A post-contrast fat-suppressed axial T1-weighted image revealing contrast enhancement in the marrow and juxtacortical soft tissue enhancement in the right tibia.

An 8-year old girl born to first-degree healthy consanguineous parents was admitted with painful swelling of the left lower leg that had lasted 10 days. Plain radiography showed a diaphyseal periosteal reaction (Figure 1a). Complete blood count was normal. Because of the absence of fever, a tumor was considered. MRI showed reduced signal intensity in the medullary bone with similar changes in the periosteum and soft tissues on T1-weighted images (Figure 1b). On T2-weighted scans, corresponding areas were hyperintense. Post-intravenous contrast images revealed enhancement of bone and adjacent soft tissues (Figure 1c). Antibiotic therapy (cephazolin sodium, 500 mg twice a day intravenously for 2 weeks) was instituted for suspected osteomyelitis. Shortly thereafter, both symptoms and radiographic findings resolved. 7 months later, the girl was re-admitted with right lower leg pain and swelling. Plain films and MRI showed similar findings to those observed previously in the left leg. Mild hyperostosis of the tibia was also noted (Figure 1d–f). Routine blood tests were normal. With a presumptive diagnosis of malignancy or recurrent osteomyelitis, the patient underwent bone biopsy, which showed reactive periostitis and normal osteoblastic cells. No organisms were identified in the aspirate, and cultures were sterile. Additional blood tests revealed increased serum phosphate (9.2 mg/dL; age-adjusted normal values: 3.6–5.9 mg/dL), normal serum creatinine, calcium, parathyroid hormone, and vitamin D levels. Serum phosphate levels were persistently high during a 12-month follow-up period. FGF23 C-terminal serum levels were elevated, as previously shown in HHS and HFTC (Topaz et al. 2004). Taken together, clinical, radiological, metabolic, and histopathologic findings suggested a diagnosis of HHS.

### Mutation analysis

**Figure 2.**
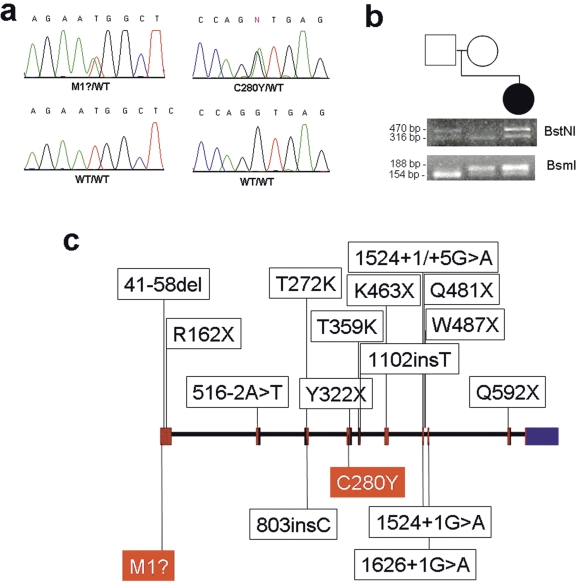
Mutation analysis. (a) Direct sequencing revealed a heterozygous T→A transversion at position 2 of the GALNT3 cDNA sequence (upper left panel) and a heterozygous G→A transition at position 839 of the GALNT3 cDNA sequence (upper right panel). The corresponding wild-type sequences are shown for comparision (lower panels). (b) To confirm mutations c.T2A and c.G839A, PCR-RFLP assays were performed. (c) GALNT3 mutations causing HFTC and HHS (respectively) are marked above and below a schematic representation of the GALNT3 gene (with the mutations identified in the present study shown in red).

HHS has recently been shown to result from mutations in the GALNT3 gene (Frishberg et al. 2005, 2007, Ichikawa et al. 2007). To confirm a diagnosis of HHS, we therefore screened genomic DNA extracted from our patient for pathogenic mutations in GALNT3. We identified two heterozygous mutations: a maternal mutation, c.T2A, predicted to disrupt the initiation codon (p.M1?); and a paternal missense mutation, c.G839A (parental origin was determined by direct sequencing of DNA samples obtained from the two parents) (Figure 2a). This last mutation is predicted to not only result in the substitution of an aromatic tyrosine residue for a well-conserved aliphatic cysteine residue (ConSeq score = 7; http://conseq.bioinfo.tau.ac.il) at amino acid position 280 (p.C280Y), but also to disrupt the consensus acceptor splice site of intron 3, leading to aberrant GALNT3 RNA processing.

We used PCR-RFLP assays to verify segregation of the mutations in the family and to exclude the 2 mutations from a panel of 50 control individuals (Figure 2b).

## Discussion

Our case remarkably illustrates the characteristic clinical and molecular features and also the diagnostic dilemmas associated with autosomal recessive HHS.

The recent identification of GALNT3 mutations in both HFTC and HHS patients (Figure 2c) (Topaz et al. 2004, Frishberg et al. 2005, 2007, Ichikawa et al. 2005, 2006, 2007, Campagnoli et al. 2006, Garringer et al. 2006, Specktor et al. 2006, Barbieri et al. 2007, Garringer et al. 2007) has revealed that these metabolic disorders result primarily from aberrant regulation of FGF23-mediated phosphaturia (Kato et al. 2006, Frishberg et al. 2007). Two explanations have been advanced to explain the fact that mutations in GALNT3 lead to 2 distinct clinical phenotypes: HHS and HFTC. Firstly, mutations affecting separate domains of the gene product may result in different phenotypes. This hypothesis is not, however, in line with the fact most mutations reported to date in GALNT3 are predicted (Topaz et al. 2004, Frishberg et al. 2005, 2007, Ichikawa et al. 2005, 2006, 2007, Campagnoli et al. 2006, Garringer et al. 2006, 2007, Specktor et al. 2006, Barbieri et al. 2007)—and in one case have been shown (Topaz et al. 2005)—to result in ppGalNacT3 deficiency. Moreover, a single founder mutation has been shown to underlie both HFTC and HHS in two inbred Israeli populations (Frishberg et al. 2005), suggesting that still unknown modifiers play a pivotal role in the pathogenesis of each disorder.

Radiological evidence of periosteal new bone formation and cortical hyperostosis are characteristically found in HHS, but they also often suggest malignancy or osteomyelitis (Talab and Mallouh 1988). A high degree of suspicion is warranted in order to avoid unnecessary procedures such as bone biopsies and nuclear scan. Imaging can be helpful to narrow the differential diagnosis in such cases. MRI can demonstrate fluid accumulation in acute osteomyelitis due to reactive edema surrounding the infection process. Chronic multifocal osteomyelitis involves the metaphyses of multiple bones, and manifest radiologically with a mixture of destructive and sclerotic areas. Langerhans cell histiocytosis shows a characteristic pattern of a lytic lesion with a benign-appearing thick periosteal reaction. The diaphysis is more commonly involved than the metaphysis (Laor and Oestreich 1998). Approximately 20% of leukemia patients experience bone pain, which is classically periarticular and migratory. Multiple, small, circumscribed osteolytic lesions often reflect leukemic involvement in bone tissue (Kobayashi et al. 2005). Typical skeletal abnormalities in metastatic neuroblastoma consist of lytic, permeative lesions with metaphyseal involvement (Kaufman et al. 1978). Primary bone tumors usually have a soft tissue component (Laor and Oestreich 1998).

In summary, despite the value of in-depth clinical and radiological evaluation in cases suspected of being HHS, today molecular diagnosis represents the best way to reach a definitive diagnosis, to avoid unnecessary interventions, and to provide affected individuals with adequate counseling.
